# Oral self-assembly nanoemulsion drives *in vivo* hepatic stellate cell-targeting drug delivery in liver fibrosis

**DOI:** 10.1080/10717544.2026.2686514

**Published:** 2026-06-12

**Authors:** Xinjing Shen, Jie Deng, Aijiao Jiang, Qianqi Cai, Deyong Tian, Linlin Zhao, Jing Ye, Lie Zhang, Yucheng Xiang, Quan Zhang

**Affiliations:** a Department of Neurosurgery, The First Affiliated Hospital of Chengdu Medical College, Chengdu, China; b Key Laboratory of Structure-Specific Small Molecule Drugs at Chengdu Medical College of Sichuan Province, School of Pharmacy, Chengdu Medical College, Chengdu, China; c Institute of Materia Medica, Chengdu Medical College, Chengdu, China; d School of Clinical Medicine, Chengdu Medical College, Chengdu, China; e Development and Regeneration Key Laboratory of Sichuan Province, School of Basic Medical Sciences, Chengdu Medical College, Chengdu, China; f Chengdu Nature's Grace Biological Technology Co., Ltd., Chengdu, China

**Keywords:** Oral delivery, Vitamin A, self-nanoemulsifying drug delivery system, hepatic stellate cell, liver fibrosis

## Abstract

Hepatic fibrosis is a chronic liver disease that requires long-term treatment and is characterized by the excessive accumulation of extracellular matrix (ECM), which is produced primarily by activated hepatic stellate cells (aHSCs). Oral medication represents a non-invasive and crucial strategy for prolonged therapy. However, achieving targeted drug delivery to aHSCs through oral administration remains a significant challenge because of the susceptibility of nanoparticles to structural disruption during intestinal transit. To address this, an oral vitamin A (VA)-functionalized self-nanoemulsifying drug delivery system (termed VA-SNEDDS) was fabricated for the precise delivery of morin (MOR) to aHSCs for the treatment of liver fibrosis. After oral administration, the designed VA-SNEDDS successfully translocated across the intestinal epithelium while maintaining the structural integrity of the nanoemulsion, entered the systemic circulation *via* the lymphatic pathway, and ultimately accumulated in aHSCs within fibrotic livers through VA- and retinol-binding protein receptor-mediated binding. In a carbon tetrachloride (CCl_4_)-induced fibrotic rat model, treatment with MOR-loaded VA-SNEDDS significantly attenuated liver fibrosis by reducing ECM deposition, hydroxyproline content, and transforming growth factor-β1 (TGF-β1) expression while concurrently restoring liver function. This study provides an orally administrable aHSCs-targeted platform with high translocation efficiency and indicates potential for this strategy in the treatment of liver fibrosis.

## Graphical abstract

**Figure:**
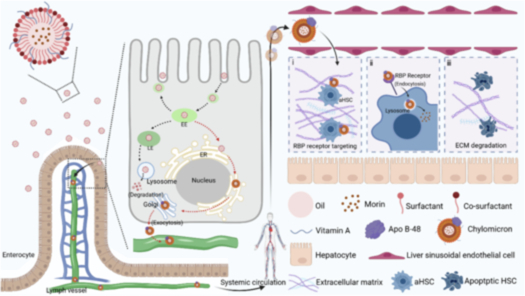

## 1. Introduction

The liver, a vital organ in human metabolism, is highly susceptible to damage from various factors, such as viral infections, alcohol, and metabolic diseases. Globally, liver diseases cause approximately two million deaths annually, accounting for 3.6% of total mortality (Mak et al. 2024; Sung et al. 2021). In most patients, liver disease progression is accompanied by worsening hepatic fibrosis, which can rapidly advance to cirrhosis or hepatocellular carcinoma in later stages, posing a serious life-threatening risk (Cheng et al. 2025; Kisseleva and Brenner 2021; Parola and Pinzani 2019). Hepatic fibrosis is a reversible and dynamic process, offering hope for therapeutic intervention (Kisseleva and Brenner 2021; Seo et al. 2020; Shi et al. 2025). To date, only rezdiffra, a thyroid hormone receptor-β (THR-β) agonist, has received approval from the United States Food and Drug Administration for the treatment of adults with nonalcoholic steatohepatitis (NASH), however, only about 26% of NASH patients show the reversal of hepatic fibrosis (Harrison et al. 2023; Kokkorakis et al. 2024). Additionally, most of current candidate drugs suffer from poor bioavailability and inadequate hepatic accumulation, thereby failing to achieve satisficed therapeutic results (Balaji et al. 2023; Wang et al. 2025). Therefore, it is urgent to develop a safe and efficacious strategy for the treatment of hepatic fibrosis.

Hepatic fibrosis is chiefly characterized by excessive deposition of extracellular matrix (ECM) following chronic liver injury (Chen et al. 2023; Tsuchida and Friedman 2017). Hepatic stellate cells (HSCs) are the predominant cellular origin of the ECM during fibrosis and thus play pivotal drivers of liver fibrogenesis (Booijink et al. 2023; Friedman 2008). Under physiological conditions, HSCs reside in a quiescent state and are located within the space of Disse (Blaner et al. 2009; Senoo et al. 2007). Upon liver injury, these quiescent HSCs become activated to synthesize and secrete excessive ECM, thereby forming fibrotic scars (Mederacke et al. 2013; Roehlen et al. 2020). Present researches indicated that the targeted regulation of activated HSCs (aHSCs) could regress hepatic fibrosis (Kisseleva et al. 2012; Lou et al. 2015; Ma et al. 2022). Nevertheless, this reparative transformation unfolds over an extended period and requires long-term and repeated treatment. Oral medication treatment is a convenient and non-invasive administration strategy that is particularly crucial for patients requiring long-term treatment (El-Mezayen et al. 2018; He et al. 2019; Ren et al. 2023).

**Figure 1.**
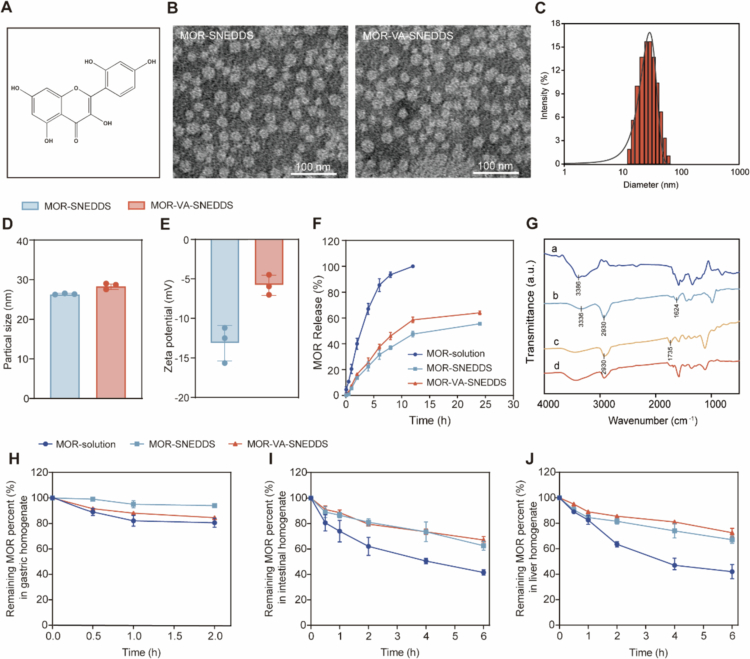
Characterization of MOR-VA-SNEDDS. (a) Chemical structure of morin. (b) Representative TEM images of MOR-SNEDDS and MOR-VA-SNEDDS. Scale: 100 nm. (c) The particle size distribution of the MOR-VA-SNEDDS. (d and e) Particle size (d) and zeta potential (e) of MOR-SNEDDS and MOR-VA-SNEDDS (*n* = 3). (f) Drug release curve of MOR in pH 7.4 phosphate-buffered solution at 37 °C (*n* = 3). (g) FTIR spectra of the MOR-VA-SNEDDS. (h–j) Stability of MOR for MOR-VA-SNEDDS in gastric (h), intestinal (i), and liver (j) homogenates (*n* = 3).

Although oral administration is widely utilized in clinical practice, the complex milieu of the gastrointestinal tract, characterized by fluctuating pH, digestive enzymes, and a protective mucus layer, can lead to drug degradation, inactivation, and poor absorption, consequently resulting in low oral drug bioavailability (Ren et al. 2023; Zeiser et al. 2022). Furthermore, for effective liver fibrosis therapy, it is crucial to facilitate the selective distribution of absorbed drugs into the fibrotic liver (Shan et al. 2024). The drug used in this study, morin (MOR, 3,5,7,2′,4′-pentahydroxyflavone, Figure 1A), is a typical bioflavonoid belonging to the flavonol subclass (Mendoza-Wilson et al. 2011; Sang et al. 2017). Owing to its multiple phenolic hydroxyl groups and conjugated double bond, MOR exhibits potent antioxidant activity. Consequently, it can reduce oxidative stress, inhibit inflammatory mediators such as tumor necrosis factor-alpha (TNF-α) and interleukin-6 (IL-6) (Lee et al. 2008; Tian et al. 2017), and protect against CCl₄-induced liver fibrosis through activation of the NF-E2-related factor 2 (Nrf2) signaling pathway (Sang et al. 2017). However, the therapeutic potential of MOR is limited by its hydrophobic skeleton, which results in low aqueous solubility and poor oral bioavailability. Therefore, there is an urgent need to develop effective strategies for an oral drug delivery system capable of targeted delivery to the liver.

Dietary triglycerides can be packaged with apolipoproteins (such as Apo B-48) into chylomicrons (CMs), which ultimately enter the bloodstream *via* the lymphatic system (Zarkada et al. 2023). Our previous studies illustrated that a self-nanoemulsifying drug delivery system (SNEDDS) could mimic CMs to be absorbed by the lymphatic pathway and protect oral drugs against degradation in the gastrointestinal tract (Wang et al. 2023; Zhang et al. 2021). Additionally, HSCs act as the major storage site for vitamin A (VA) and express high levels of the retinol-binding protein (RBP) receptor, which is known for its specific binding to VA (Haaker et al. 2024; Qiao et al. 2018). This high level of receptor expression is crucial for efficient VA uptake, and the hydrophobic nature of free VA necessitates its transport in the bloodstream bound to plasma RBP, facilitating its delivery to cells *via* the stimulated by the retinoic acid 6 (STRA6) membrane receptor. However, studies indicate that RBP may not be strictly essential for VA uptake by HSCs. VA may directly interact with other VA-binding proteins on the cell membrane to activate cellular uptake mechanisms (Blaner et al. 2016; El-Mezayen et al. 2017).

Inspired by these findings, we fabricated a VA-functionalized SNEDDS (VA-SNEDDS) by incorporating VA into SNEDDS to deliver MOR to HSCs for hepatic fibrosis treatment. Following oral administration, the designed VA-SNEDDS was absorbed into systemic circulation *via* the lymphatic transport pathway, a route that bypassed hepatic first-pass metabolism and contributes to enhanced oral bioavailability. Furthermore, the absorbed VA-SNEDDS would exploit the high-affinity interaction between the VA incorporated into the nanoemulsion surface and the receptors on aHSCs to achieve selective drug delivery within the fibrotic liver. In a CCl_4_-induced fibrotic rat model, treatment with MOR-loaded VA-SNEDDS (MOR-VA-SNEDDS) significantly ameliorated the progression of liver fibrosis. In summary, this study presents an innovative, convenient, and efficacious oral liver-targeting drug delivery strategy for the treatment of hepatic fibrosis, offering valuable insight and reference for the oral liver-targeting strategy.

## 2. Materials and methods

### 2.1. Materials and animals

Morin (purity > 98%) was acquired from Jiangsu Aikon Biopharmaceutical R&D Co., Ltd. (Jiangsu, China). Cremophor RH 40 was sourced from Shandong Keyuan Biochemical Co., Ltd. (Shandong, China). Labrafil M 1944CS was sourced from Shanghai Yuanye Biotechnology Co., Ltd. (Shanghai, China). Transcutol HP, vitamin A (VA, retinol) and tilaurin were sourced from Shanghai Macklin Biochemical Technology Co., Ltd. (Shanghai, China). 1,1′-Diethyl-3,3,3′,3′-tetramethylindocarbocyanine (DiD), 3,3′-dioctadecyloxacarbocyanine perchlorate (DiO) and 1,1′-dioctadecyl-3,3,3′,3′-tetramethylindocarbocyanine perchlorate (DiI) were sourced from Shanghai Macklin Biochemical Technology Co., Ltd. (Shanghai, China). Cycloheximide (CYC) was sourced from Chengdu Huaxia Chemical Reagent Co., Ltd. (Chengdu, China). The Caco-2 cell line was purchased from the American Type Culture Collection. Primary rat HSCs were acquired from Xiamen Immocell Biotechnology Co., Ltd. (catalog number: IMP-R041). The RBP4 protein Rat (HEK293, His) was obtained from MedChemExpress LLC (China). Dulbecco's modified Eagle's medium (DMEM, high-glucose), fetal bovine serum (FBS), Hanks' balanced salt solution (HBSS) and penicillin‒streptomycin solution were purchased from Thermo Fisher Scientific, Inc. (Waltham, USA). All other chemicals and solvents were analytical or chromatographic grade.

A total of eighty-four healthy male Sprague–Dawley (SD) rats (200 ± 20 g, 5 weeks old) were used in this work. All the animals were obtained from Chengdu Dashuo Laboratory Animal Co., Ltd. (Chengdu, China), and the Animal License Number was SCXK(川)2020-0030. The animals were housed under specific pathogen-free (SPF) conditions at a standard temperature of 25 °C ± 2 °C and a relative humidity of 75% ± 5%, with free access to standard food and water. The animal experimentation began on November 25, 2023, and ended on July 30, 2024. All animal studies were conducted in accordance with the requirements of the National Regulations on the Management of Experimental Animals (China) and adhered to the guidelines evaluated and approved by the Animal Ethics Committee of Chengdu Medical College (Approval NO. CMC-IACUC-2023090).

The use of animals was justified by the need to evaluate the antifibrotic efficacy and targeting of the tested formulations within a physiologically relevant context. While *in vitro* models serve as valuable tools for preliminary screening, they are unable to reflect intricate multicellular interactions, the dynamic processes of extracellular matrix deposition and resolution, as well as the drug targeting, systemic pharmacokinetics, drug distribution, and *in vivo* antifibrotic efficacy characteristics that are essential for evaluating therapies aimed at hepatic fibrosis. Furthermore, the rat CCl₄ model is among the most widely employed methods for investigating the mechanisms underlying liver fibrosis. (Starkel and Leclercq 2011; Tsuchida and Friedman 2017).

### 2.2. Preparation of MOR-VA-SNEDDS

Based on our previous research (Zhang et al. 2011), we established the formulation of the blank excipient SNEDDS and confirmed the proportional formulation of the preparation accordingly. Two hundred milligrams of Labrafil M 1944CS, 350 mg of Cremophor RH 40, and 450 mg of Transcutol HP were vortex-mixed thoroughly to obtain blank SNEDDS. Then, 25 mg of MOR was dispersed into the blank SNEDDS, followed by vortexing until complete dissolution to obtain the MOR-SNEDDS. A total of 3 mg of VA was added to the blank SNEDDS and vortex-mixed thoroughly to prepare VA-SNEDDS. Then, 25 mg of MOR was accurately weighed and dispersed into the VA-SNEDDS, followed by vortexing until complete dissolution to obtain MOR-VA-SNEDDS.

### 2.3. Characterization of MOR-VA-SNEDDS

#### 2.3.1. Encapsulation efficiency (EE) and drug loading capacity (DL)

The prepared MOR-VA-SNEDDS (0.5 mL) were dispersed in 9.5 mL of distilled water to form oil-in-water emulsions. These emulsions were then transferred to ultrafiltration centrifuge tubes (molecular weight cutoff: 3000 Da) and centrifuged at 10,000 rpm for 10 min (Wu et al. 2019). The resulting filtrate was collected from the lower compartment and subjected to high-performance liquid chromatography (HPLC, Agilent 1260 series, USA) analysis for MOR content determination. The EE and DL were calculated according to the following equations:

$$\mathrm{EE}\;\left(\%\right)=\left(1-\frac{m_{\mathrm{free}}}{m_{\text{drug}}}\right)\times 100\;\%,$$

$$\mathrm{DL}\;\left(\%\right)=\frac{m_{\mathrm{drug}}-m_{\mathrm{free}}}{m_{\mathrm{drug}}\;+m_{\mathrm{excipient}}}\times 100\;\%.$$

*m*_free_: the mass of MOR in the filtrate. *m*_drug_: the mass of MOR in MOR-VA-SNEDDS. *m*_excipient_: the total mass of excipients in MOR-VA-SNEDDS.

#### 2.3.2. *In vitro* drug release behavior

One milliliter of MOR-VA-SNEDDS (with a MOR concentration of 1 mg/mL) was added into a dialysis bag (molecular weight cutoff: 7800 Da). The dialysis bag was then placed in a sterile glass bottle containing 50 mL of release medium (phosphate-buffered saline (PBS), pH 7.4). The glass bottle was positioned in a horizontal thermostatic shaker set at 37 °C with an oscillation speed of 100 rpm. At predetermined time intervals, 1 mL of the release medium was withdrawn and replaced with an equal volume of fresh release medium. The MOR content in the collected samples was quantified using HPLC.

#### 2.3.3. Fourier-transform infrared (FTIR) spectra

Approximately 10 mg of MOR-VA-SNEDDS was mixed with potassium bromide and compressed into a pellet. The pellet was then analyzed using a FTIR spectrometer (Thermo Scientific Nicolet iS20, USA) over a wavelength range of 500–4000 cm^−1^.

#### 2.3.4. Particle size and zeta potential

The particle size and zeta potential of the MOR-SNEDDS and MOR-VA-SNEDDS were determined using a laser particle analyzer (MalvernZS-90, Worcestershire, UK). Each formulation was diluted 100-fold with distilled water and emulsified under stirring in a 37 °C water bath. The emulsified sample was then transferred into a measurement cell, and measurements were performed in triplicate at 25 °C.

#### 2.3.5. Stability of MOR in tissue homogenates

The stability of MOR from MOR-DMSO, MOR-SNEDDS, and MOR-VA-SNEDDS was assessed in stomach, intestinal, and liver tissue homogenates. Each blank homogenate (900 μL) was mixed with 100 μL of the respective formulation and incubated at 37 °C. At the designated time points, 100 μL aliquots were collected and mixed with 500 μL methanol followed by vortexing for 10 min and centrifugation at 12,000 rpm for 10 min at 4 °C. The resulting supernatant was filtered through a 0.22 μm membrane and subjected to HPLC.

### 2.4. *In vitro* cell tests

#### 2.4.1. Cells

In June 2022, the Caco-2 cell line was obtained from the American Type Culture Collection (catalog number: HTB-37), and the HSC-T6 cell line was acquired from Pricella Life Science & Technology Co., Ltd. (catalog number: CL-0116). The Caco-2 cell line was authenticated by short tandem repeat (STR) profiling immediately upon receipt and tested negative for mycoplasma contamination using a polymerase chain reaction (PCR)-based assay. All Caco-2 cells were used within 15 passages after resuscitation and were routinely checked for mycoplasma contamination every month.

#### 2.4.2. Cellular uptake

HSC-T6 cells, a type of HSCs, were seeded in confocal dishes at a density of 2.0 × 10⁵ cells/well and exposed to TGF-β (10 ng/mL) for 24 h to trigger their activation. The cells were then treated with DiD-labeled VA-SNEDDS (DiD-VA-SNEDDS, DiD concentration: 500 ng/mL) in serum-free medium for 1 h, followed by three washes with PBS. The cells were subsequently fixed with paraformaldehyde (4%) for 10 min and washed three times again. Under light-protected conditions, 4′,6-diamidino-2-phenylindole dihydrochloride (DAPI) working solution (1.5 μg/mL) was added, and incubated at 37 °C for 15 min for nuclear staining. Finally, the cells were washed three times with PBS and visualized under an confocal microscopy (Olympus FV1000 series, Japan). Cellular uptake by primary rat HSCs was performed using the same protocols as those described above for HSC-T6 cells.

#### 2.4.3. Cell apoptosis assay

The activated HSC-T6 cells by TGF-β were treated with free MOR or MOR-loaded nanoemulsions for 12 h. The treated cells were washed with PBS and resuspend in binding buffer. Then, fluorescein isothiocyanate (FITC)-labeled Annexin V and propidium iodide (PI) were added and thoroughly mixed. The mixture was incubated at room temperature in the dark for 10 min and subsequently analyzed using a flow cytometry (Thermo Fisher Attune NxT, USA), with Annexin V-FITC and PI signals detected in the FL1 and FL3 channels, respectively. The cell apoptosis assay in primary rat HSCs was performed using the same protocols as those described above for HSC-T6 cells.

#### 2.4.4. Determination of fibrosis markers

The activated HSC-T6 cells by TGF-β were treated with free MOR or MOR-loaded nanoemulsions for 12 h. Then these treated cells were harvested for protein extraction. The obtained protein samples are used to determine the expression levels of fibrosis markers, such as type I collagen (collagen-I) and α-smooth muscle actin (α-SMA) through Western blot (WB) assay. The WB bands were semi-quantified by the Image J. The pre-stained protein molecular weight marker (catalog number: G2087-250UL) from Wuhan Servicebio Technology Co., Ltd. was used in all the WB experiments.

#### 2.4.5. Competitive cellular uptake inhibition assay

Primary rat HSCs were seeded in confocal dishes at a density of 2.0 × 10⁵ cells/well and exposed to TGF-β (10 ng/mL) for 24 h to trigger their activation. The cells were then pre-treated with either free VA (0.7 μg/mL) or RBP4 (0.7 μg/mL) for 1 h prior to incubation with DiD-labeled nanoparticles (DiD-SNEDDS, DiD-VA-SNEDDS, DiD concentration: 500 ng/mL). After 4 h of incubation, the cells were washed three times with PBS. Then the cells were fixed with paraformaldehyde (4%) for 10 min and washed three times again. Under light-protected conditions, DAPI working solution (1.5 μg/ml) was added, and incubated at 37 °C for 15 min for nuclear staining. Finally, the cells were washed three times with PBS and observed under a confocal microscope (Olympus FV1000 series, Japan). Fluorescence scanning of the cell images was performed using ImageJ for quantitative analysis.

#### 2.4.6. Cytotoxicity assay

Caco-2 cells were seeded in 96-well plates at 5 × 10⁴ cells/well and incubated for 24 h. The cells were treated with various concentrations of VA-SNEDDS, free MOR, or MOR-VA-SNEDDS (MOR concentration: 1, 5, 10, 25, and 50 µg/mL) in complete medium for 12 h. Following treatment, 10 µL of 3-(4,5-dimethylthiazol-2-yl)-2,5-diphenyltetrazolium bromide (MTT) solution (5 mg/mL) was added to each well, and the plates were incubated at 37 °C for 4 h in the dark. Then, 150 µL of DMSO was added to each well, followed by incubation with shaking at 37 °C for 10 min. Subsequently, thoroughly dissolve the blue methylene blue crystals in each well with 150 µL of DMSO. The absorbance was measured at 490 nm using a microplate reader (Flash Spectrum, China), with untreated cells serving as the control and cell-free wells used for zero adjustment. All the treatments were tested in triplicate. Cell viability (%) was calculated as:

$$\mathrm{Cell}\;\mathrm{viability}\;\left(\%\right)=\frac{\mathrm{OD}\;\mathrm{of}\;\mathrm{sample}-\mathrm{OD}\;\mathrm{of}\;\mathrm{blank}}{\mathrm{OD}\;\mathrm{of}\;\mathrm{control}-\mathrm{OD}\;\mathrm{of}\;\mathrm{blank}}.$$

#### 2.4.7. Caco-2 cell monolayer

Caco-2 cells were seeded at a density of 2 × 10^5^ cells/well in Transwell inserts (12-well). The upper chamber of the Transwell system was filled with 1.5 mL of DMEM medium containing cells, while the lower chamber was supplemented with 2.6 mL of cell-free DMEM medium. During the first week after seeding, the medium was replaced every other day, and thereafter, it was refreshed daily. The cells were cultured for three weeks, and Transwell membranes meeting the criterion of transepithelial electrical resistance (TEER) > 800 Ω·cm^2^ were selected for subsequent drug transport experiments.

#### 2.4.8. Transport of VA-SNEDDS across the Caco-2 cell monolayer

For the transport study, 1.5 mL of HBSS containing MOR-VA-SNEDDS (MOR concentration: 50 μg/mL) was added to the apical side of the Transwell device, while 2 mL of blank HBSS was added to the basolateral side. The system was incubated for 4 h in the CO₂ incubator. Subsequently, the liquid samples collected from the apical and basolateral sides were subsequently diluted 10 times with deionized water, spotted onto copper mesh and stained with 1% phosphotungstic acid, and the staining solution was gently absorbed with filter paper. The samples were then observed and photographed using a transmission electron microscope (TEM, Hitachi H-600, Japan). Additionally, HBSS containing MOR-VA-SNEDDS or triglyceride (TG) was added into the apical side of the Transwell device. After incubation for 24 h, the HBSS from the basolateral sides was collected to detect Apo B-48 through WB analysis.

To verify whether VA-SNEDDS could maintain structural integrity during transcellular transport, 1.5 mL of DiO/DiI-VA-SNEDDS in HBSS (dye concentration: 500 ng/mL) was added to the apical side of the Transwell device, while 2 mL of blank HBSS buffer was added to the basolateral sides. The Transwell device was then incubated in a 37 °C incubator for 4 h. After incubation, liquid samples were collected separately from both sides and subjected for fluorescence microscopy observation (Nikon ECLIPSE Ti, Japan). DiO and DiI signals were detected using the FITC and Cy3 channels, respectively.

#### 2.4.9. Measurement of sodium fluorescein permeability

Following the transport study, the apical and basolateral chambers were washed three times with pre-warmed HBSS to eliminate any residual SNEDDS. Subsequently, 1 mL of sodium fluorescein solution (20 µg/mL in HBSS) was added to the apical chamber, and 2 mL of blank HBSS was added to the basolateral chamber. The fluorescence intensity was measured at 0.5, 1, and 2 h. The concentration of sodium fluorescein in the basolateral chamber was determined using a pre-established standard curve. The apparent permeability coefficient (Papp) of sodium fluorescein from the apical to the basolateral side was then calculated using the transport formula. The integrity of the Caco-2 cell monolayers was considered maintained when the TEER of each insert exceeded 800 Ω·cm² and the Papp of the low-permeability marker sodium fluorescein was less than 1 × 10^−6^ cm/s. The Papp was calculated according to the following equation:

$$P_{\mathrm{app}}=\frac{dQ}{dT}\times \frac{1}{A\times C_{0}}.$$

d*Q*/d*T*: the rate of change in the amount transported per unit time (µg/s). *A*: the effective surface area of the membrane (cm²). *C*_0_: the initial concentration of the drug (µg/mL).

#### 2.4.10. Plasma localization of VA-SNEDDS

Six normal SD rats were randomly divided into 2 groups and orally administered with DiO/DiI-VA-SNEDDS or the mixture of DiO-VA-SNEDDS and DiI-VA-SNEDDS (500 μg dye/kg). The rats were euthanized at 15 min post-administration, and blood samples were collected for fluorescence microscopy observation (Nikon ECLIPSE Ti, Japan). DiO and DiI signals were detected using the FITC and Cy3 channels, respectively.

### 2.5. Liver fibrosis model

Rats were intraperitoneally injected with carbon tetrachloride (CCl_4_) dissolved in olive oil at a volume ratio of 4:6 (v/v). The administration dose was 1.5 mL/kg body weight, with the initial dose doubled. This treatment was performed three times per week for six consecutive weeks.

### 2.6. *In vivo* imaging

DiD was utilized as the tracer for *in vivo* imaging in model animals. Nine fibrotic rats were randomly divided into three groups. Each group was orally administered free DiD, DiD-SNEDDS, or DiD-VA-SNEDDS (2 mg DiD/kg) respectively. The *in vivo* imaging of the rats was conducted at 2-, 6-, and 24-h post-administration through an *in vivo* imaging sys-tem (Revvity IVIS Lumina III, USA). After 24 h, the rats were euthanized and subjected to *ex vivo* imaging of their respective organs. DiD signals from both *in vivo* and *ex vivo* images were detected *via* the Cy5 channel. Additionally, six fibrotic rats were orally treated with DiD-SNEDDS, or DiD-VA-SNEDDS (2 mg DiD/kg) respectively. Liver tissues were collected to prepare sections 4 h after administration. The prepared sections of 10 μm thickness were stained with a rat platelet-derived growth factor-β (PDGFR-β) antibody (CST, USA). DAPI was applied to stain the cell nuclei. The fluorescent distributions in liver tissues were observed with a fluorescence microscope (Pannoramic MIDI, 3DHISTECH, Hungary) using the FITC channel.

### 2.7. Intestinal fluorescence staining

Oral administration of DiD-VA-SNEDDS (at a DiD dose of 2 mg/kg) was given to three rats, and the rats were then euthanized at 15 min after dosing. The duodenum, jejunum, and ileum segments were isolated for frozen sectioning. The sections were mounted on glass slides and fixed with 4% paraformaldehyde. A donkey serum albumin solution was applied to the slide sections and incubated at 37 °C in an air-bath shaker for 30 min. After washing off the albumin solution, FITC-phalloidin was added to stain F-actin in the tissue sections. The slides were incubated in the dark at room temperature for 50 min, and the excess dye was rinsed off with PBS. Next, DAPI was used to stain the cell nuclei, and the slides were sealed with an anti-fade mounting medium. The intestinal tissue sections were observed and imaged under a fluorescence microscope (Pannoramic MIDI, 3DHISTECH, Hungary) using the FITC channel.

### 2.8. Pharmacokinetics study

Twenty rats were evenly divided into 4 groups. They were orally administered with MOR suspension, MOR-SNEDDS, MOR-VA-SNEDDS, or MOR-VA-SNEDDS + CYC at a dose equivalent to 100 mg/kg of MOR. Rats in the MOR-VA-SNEDDS + CYC group were intraperitoneally injected with a solution of CYC (3 mg/kg) one hour prior to the experiment. Blood samples (0.3 mL) were collected from the retro-orbital venous plexus at predetermined time intervals into 1.5 mL centrifuge tubes. The samples were centrifuged at 5000 rpm for 10 min at 4 °C, and 100 μL of the supernatant plasma was collected. Subsequently, 500 μL of methanol was added, followed by vortex for 10 min and centrifugation at 12,000 rpm for another 10 min at 4 °C. The resulting supernatant was analyzed *via* a high-performance liquid chromatography/mass spectrometer triple quadrupole system (HPLC-MS/MS, Agilent Triple Quad 6410, USA) to determine the plasma concentration of MOR. Chromatographic separation was performed on an Agilent XDB C18 column (4.6 × 50 mm, 1.8 μm) maintained at 35 °C using an isocratic mobile phase of water:methanol (45:55, v/v) at a flow rate of 0.1 mL/min, and the injection volume was 1 μL. The triple quadrupole mass spectrometer was operated in negative electrospray ionization (ESI) mode with multiple reaction monitoring (MRM). The transition for MOR was monitored at m/z 301 → 151. The optimized MS/MS parameters were as follows: capillary voltage, 4 kV; nebulizer gas pressure, 15 psi; drying gas (N₂) temperature, 350 °C; drying gas flow, 11 L/min; fragmentor voltage, 145 V; and collision energy, 17 V.

### 2.9. *In vivo* biodistribution assay

Fifteen rats were evenly divided into 3 groups. They were orally administered with MOR suspension, MOR-SNEDDS, or MOR-VA-SNEDDS (100 mg MOR/kg). After 30 min, the rats were euthanized, and the organs (heart, liver, spleen, lung, and kidney) were excised, weighed, and placed in 2 mL centrifuge tubes. A volume of physiological saline equivalent to three times the tissue weight was added to prepare tissue homogenates. A 100 μL aliquot of the homogenate was mixed with 400 μL of methanol, vortexed for 10 min, and centrifuged at 12,000 rpm for 10 min at 4 °C. The supernatant was subjected to HPLC-MS/MS analysis to quantify the MOR content in the main organs.

### 2.10. *In vivo* anti-fibrotic efficacy

To evaluate the anti-fibrotic activity of MOR-VA-SNEDDS *in vivo*, twenty rats with liver fibrosis were divided into four groups and orally administered with saline, MOR suspension, MOR-SNEDDS, and MOR-VA-SNEDDS, respectively. Normal rats, as a control, were orally given with saline. The treatments were administered once daily for 4 weeks. At the end of the experiment, the major organs were dissected, and the tissue samples were preserved in 10% neutral buffered formalin for subsequent analysis.

### 2.11. Quantitative assessment of liver fibrosis

The serum biomarkers of liver injury, including alanine aminotransferase (ALT), aspartate aminotransferase (AST), and albumin (ALB), were measured with enzyme-linked immunosorbent assay (ELISA) kits following the instructions provided by the manufacturer (Beijing Solarbio Science & Technology Co., Ltd., China). Additionally, the concentrations of hydroxyproline (HYP) and transforming growth factor-β1 (TGF-β1) in liver tissue were also analyzed using ELISA kits (Elabscience Biotechnology Co., Ltd, China), adhering to the manufacturer's protocols.

### 2.12. Statistical analysis

Experimental data were subjected to statistical analysis using Prism software version 9.5 (GraphPad Software, USA). All the results were expressed as mean ± standard deviation (mean ± SD). One-way analysis of variance (ANOVA) was employed to assess the statistical significance among groups, followed by multiple comparison analysis to evaluate differences between individual groups.

## 3. Results and discussion

### 3.1. Characterization of MOR-VA-SNEDDS

Enhancing the bioavailability of hydrophobic drugs is of paramount importance, and encapsulating these agents within small-sized, highly stable nanocarriers can significantly improve the therapeutic efficacy. Morphological analysis *via* TEM imaging revealed that the emulsified droplets formed by MOR-SNEDDS and MOR-VA-SNEDDS exhibited a near-spherical shape (Figure 1B) with a uniform particle size distribution (Figure 1C). The average droplet sizes of the emulsified MOR-SNEDDS and MOR-VA-SNEDDS were 26.56 ± 0.09 nm and 28.24 ± 0.15 nm, with zeta potentials of −13.8 ± 1.94 mV and −5.48 ± 0.71 mV, respectively (Figure 1D and E). Both formulations feature particle sizes under 100 nm, which are conducive to intestinal lymphatic absorption, bypassing the hepatic portal vein and thereby mitigating the first-pass metabolism effect (Mahmoudian et al. 2020). Furthermore, the slightly lower zeta potential of the MOR-VA-SNEDDS compared to MOR-SNEDDS was helpful to increase its oral bioavailability because the neutral or mildly negative charge for nanoparticles is suited for penetrating the gastrointestinal mucus barrier and achieving systemic absorption (Gamazo et al. 2015; McCright et al. 2022). The EE of MOR in the MOR-SNEDDS and MOR-VA-SNEDDS were 98.30% and 98.51%, respectively (Table 1). In addition, the *in vitro* release results showed that the MOR solution released the drug completely within 12 h, while the drug release amounts for MOR-SNEDDS and MOR-VA-SNEDDS were 56.3% and 65.0%, respectively (Figure 1F). FTIR spectral analysis revealed that the characteristic peak of MOR appeared at 3386 cm^−1^, corresponding to the stretching vibration of its O–H group. The characteristic absorption peaks of VA at 3336 cm^−1^, 2930 cm^−1^, and 1624 cm^−1^ indicated the presence of O–H stretching, C–H stretching, and C = C stretching, respectively. The excipient VA-SNEDDS showed characteristic absorption peaks at 2930 cm^−1^ and 1753 cm^−1^, corresponding to C–H stretching and C = O stretching (Figure 1G). The FTIR spectrum of the MOR-VA-SNEDDS exhibited the same band characteristics as those of the drug and excipients, with no significant shifts in peak positions and no new peaks appearing. These results indicated that MOR was compatible with all the excipients in the formulation.

**Table 1.** EE and DL of MOR-SNEDDS and MOR-VA-SNEDDS (*n* = 3).

Formulations	EE (%)	DL (%)
MOR-SNEDDS	98.30 ± 0.37	2.37 ± 0.04
MOR-VA-SNEDDS	98.51 ± 0.25	2.32 ± 0.02

The stability of MOR throughout the absorption process is crucial for its oral bioavailability. Therefore, the stability of MOR for MOR-VA-SNEDDS was determined in the simulating gastrointestinal fluids and tissue homogenates (including stomach, intestine, and liver). As shown in Figure 1H, MOR exhibited good stability in gastric homogenate but was prone to degradation in intestinal and hepatic homogenates, with degradation rates of 57.0% and 58.4%, respectively. In contrast, MOR within MOR-SNEDDS and MOR-VA-SNEDDS remained stable in the gastric homogenate. After 6 h of incubation in intestinal homogenate, their drug content decreased by 33.4% and 27.7%, respectively (Figure 1I), while in the hepatic homogenate, the reductions were 32.7% and 27.7%, respectively (Figure 1J). These findings indicate that MOR-VA-SNEDDS significantly enhance the gastrointestinal stability of MOR. This improvement may be attributed to the formation of oil-in-water emulsions upon interaction with gastric and intestinal fluids, where the oil phase acts as a protective barrier between digestive enzymes and MOR, preventing enzymatic degradation. Furthermore, the MOR-VA-SNEDDS demonstrated partial resistance to hepatic microsomal drug metabolism, thereby improving MOR stability in the liver homogenate. This protective effect likely contributes to enhanced systemic bioavailability by reducing first-pass metabolism (Zhao et al. 2019).

### 3.2. *In vitro* antifibrotic effect of MOR-VA-SNEDDS

#### 3.2.1. The antifibrotic effect of MOR-VA-SNEDDS in HSC-T6 cells

**Figure 2.**
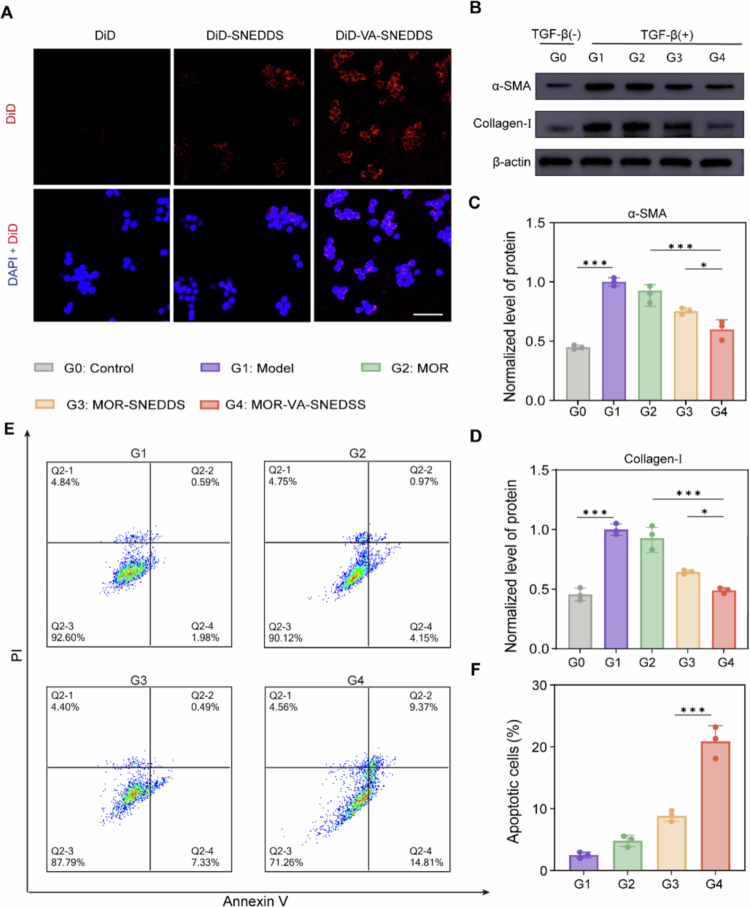
*In vitro* antifibrotic effects of MOR-VA-SNEDDS in HSC-T6 cells. (a) Confocal microscopy images of HSC-T6 cells co-incubated with DiD-VA-SNEDDS for 1 h. Scale: 50 μm. (b) WB analysis of α-SMA and collagen-I expression levels in HSCs after treatment with MOR-VA-SNEDDS for 12 h. (c and d) Semi-quantitative analysis of and collagen-I (c) and α-SMA (d) levels by the Image J (*n* = 3). (e and f) The apoptosis rate of HSC-T6 cells treated with MOR-VA-SNEDDS for 12 h detected by flow cytometry (*n* = 3). The values represent mean ± SD; *p < 0.05, ***p < 0.001.

In order to evaluate the antifibrotic effect of MOR-VA-SNEDDS *in vitro*, we first investigated its endocytosis efficiency in HSC-T6 cells. As shown in Figure 2A, the DiD-VA-SNEDDS displayed a significantly higher endocytosis compared with the DiD-SNEDDS group and the free DiD group. This result indicates that VA modification can significantly improve the specific uptake efficiency of nanoemulsions in aHSCs, providing strong experimental evidence for its potential as an aHSCs-targeting delivery carrier.

Numerous studies have confirmed the involvement of the TGF-β/Smad3 pathway in the progression of liver fibrosis and that TGF-β is a key activator of HSCs (Ceglia et al. 2016; Xu et al. 2016). As shown in Figure 2B–D, the expression of collagen-I and α-SMA in HSC-T6 cells was obviously elevated after incubation with TGF-β. The blank VA-SNEDDS showed a negligible effect on the expression of these proteins. Treatment with various MOR formulations reduced the expression of these fibrosis markers to different degrees. Notably, MOR-VA-SNEDDS treatment resulted in the most reduction for fibrosis markers among all the treatment groups (*p* < 0.05). These results suggest that VA modification enables the nanoemulsions to target aHSCs, thereby effectively suppressing the expression of collagen-I and α-SMA. Additionally, an Annexin V/PI apoptosis assay was performed to further investigate the impact of the nanoemulsions on HSCs apoptosis. The results revealed that the apoptosis rate in the MOR-VA-SNEDDS-treated group (20.88 ± 2.09%) was significantly higher than that in the VA-SNEDDS group (3.62 ± 0.76%, *p* < 0.001) and the MOR group (8.84 ± 0.72%, *p* < 0.001) (Figure 2E and F). Taken together, MOR-VA-SNEDDS could be selectively uptaken by activated HSC-T6 cells and then trigger the effective cell apoptosis and low expression of fibrosis markers.

#### 3.2.2. The ability of VA-SNEDDS to target aHSCs

**Figure 3.**
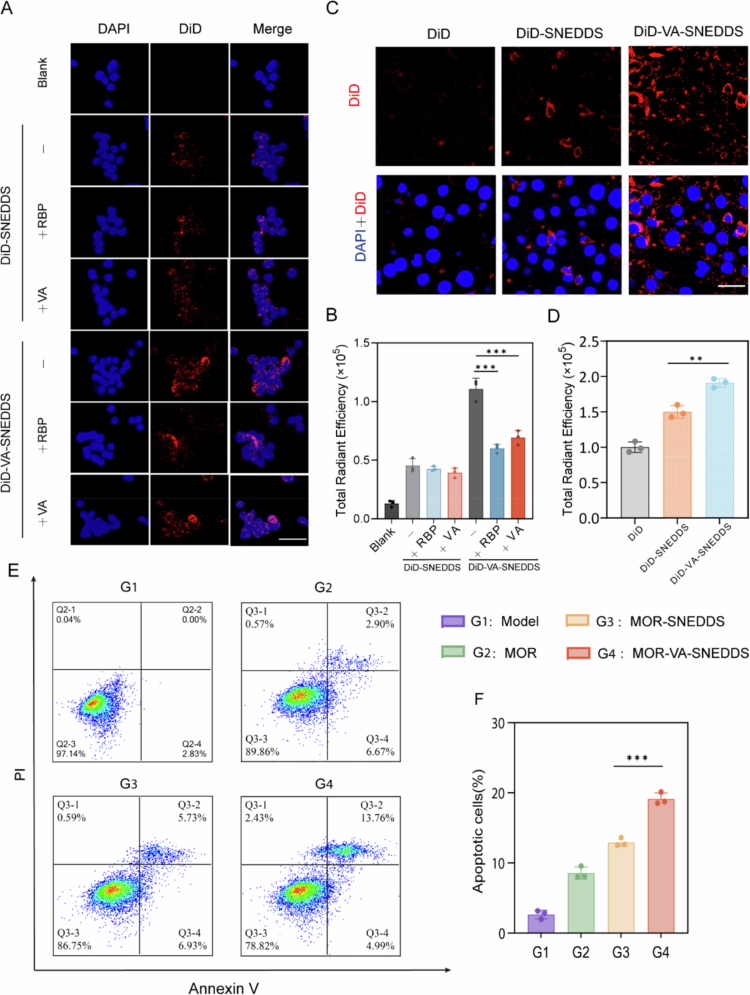
*In vitro* targeting of MOR-VA-SNEDDS to primary rat HSCs. (a) Confocal microscopy images of primary rat HSCs incubated in the culture medium containing DiD-VA-SNEDDS without/with RBP (0.7 μg/mL) or VA (0.7 μg/mL). Scale: 50 μm. (b) Semi‑quantitative analysis of the cellular fluorescence intensity treated with DiD‑VA‑SNEDDS under different competitive conditions (*n* = 3). (c) Confocal microscopy images of primary rat HSCs co-incubated with DiD-VA-SNEDDS for 1 h. Scale: 20 μm. (d) Semi‑quantitative analysis of the cellular fluorescence intensity after co‑incubation with DiD‑VA‑SNEDDS (*n* = 3). (e and f) The apoptosis rate of primary rat HSCs treated with MOR-VA-SNEDDS for 12 h detected by flow cytometry (*n* = 3). The values represent mean ± SD; **p < 0.01, ***p < 0.001.

To evaluate the potential of VA-SNEDDS in targeting aHSCs, we conducted competitive cellular uptake inhibition experiments. The results demonstrated that the cellular uptake of DiD-VA-SNEDDS in aHSCs was significantly stronger than that of the unmodified DiD-SNEDDS. However, this uptake process could be markedly inhibited by pre-incubation with free VA or RBP4 (Figure 3A). Semi-quantitative fluorescence analysis further confirmed the highest uptake efficiency in the VA-SNEDDS group, which was effectively blocked by either VA or RBP4 pre-treatment (*p* < 0.001) (Figure 3B). These findings indicate that VA-SNEDDS can effectively exploit highly expressed VA- or RBP-related recognition mechanisms on the surface of aHSCs for targeted delivery. Its uptake may involve both the classical STRA6 receptor pathway and direct interaction with other VA-binding proteins on the cell membrane.

#### 3.2.3. The antifibrotic effect of MOR-VA-SNEDDS in primary rat HSCs

Consistent with the results obtained in HSC-T6 cells, primary rat HSCs also exhibited significantly enhanced intracellular fluorescence after treatment with DiD-VA-SNEDDS, compared to DiD-SNEDDS and free DiD (Figure 3C). Flow cytometric analysis further demonstrated the potent pro-apoptotic effect of MOR-VA-SNEDDS in primary HSCs (Figure 3D and E). The apoptosis rate in the MOR-VA-SNEDDS-treated group reached 18.75%, which was significantly higher than that induced by MOR-SNEDDS (12.66%, *p* < 0.01) or free MOR (9.57%, *p* < 0.001).

### 3.3. Oral absorption mechanism of VA-SNEDDS

#### 3.3.1. Cytotoxicity of MOR-VA-SNEDDS

**Figure 4.**
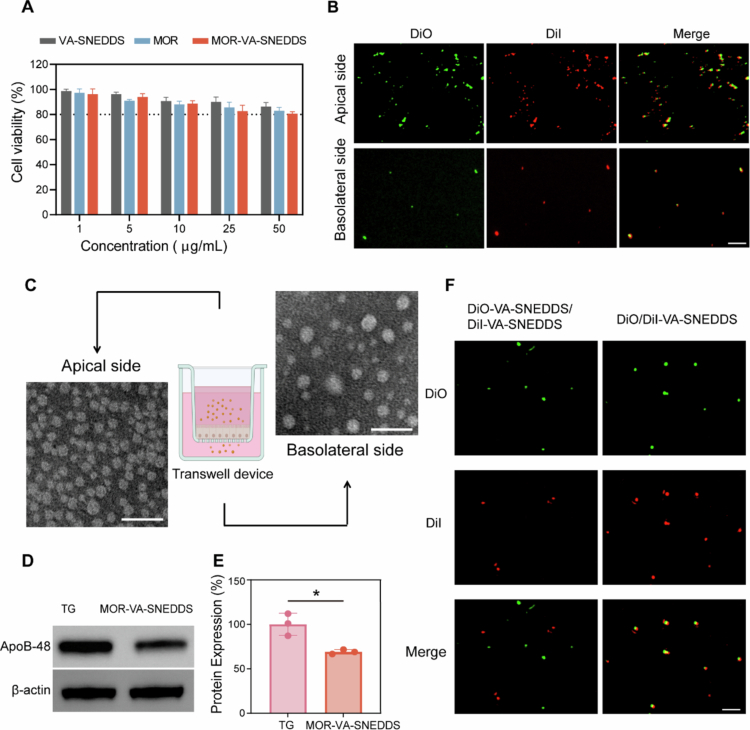
Oral absorption mechanism of MOR-VA-SNEDDS. (a) Cytotoxicity of VA-SNEDDS, MOR, and MOR-VA-SNEDDS on Caco-2 cells after incubation for 12 h (*n* = 3). (b) Fluorescent localization of DiO/DiI-VA-SNEDDS in the apical or basolateral HBSS buffer after 2 h incubation on the apical side of the Caco-2 cell monolayer. Scale: 20 μm. (c) TEM images of MOR-VA-SNEDDS on the apical and basolateral sides. Scale: 100 nm. (d) WB analysis of ApoB-48 in the basolateral HBSS buffer. (e) Semi-quantitative analysis of WB results by ImageJ (*n* = 3). (f) Fluorescent colocalization of DiI and DiO in the blood following oral administration of DiO/DiI-VA-SNEDDS. Scale: 20 μm. The values represent mean ± SD; *p < 0.05, **p < 0.01, ***p < 0.001.

The cytotoxicity of the formulations was assessed using the MTT assay on Caco-2 cells. As shown in Figure 4A, treatment with various concentrations of VA-SNEDDS, free MOR, or MOR-VA-SNEDDS for 12 h did not induce significant cytotoxicity. The cell viability remained above 80% across all the tested concentrations. Based on these results, we determined a dosing concentration range of 1–50 μg/mL to ensure no apparent toxicity to cells during the experimental period (<12 h), and all subsequent *in vitro* experiments were conducted within this safe dosage range.

#### 3.3.2. Transport of VA-SNEDDS across the Caco-2 cell monolayer

The preservation of nanoparticle integrity after crossing the intestinal epithelial monolayer is critical to harnessing their benefits for drug protection and targeted delivery (Davis et al. 2010). To accurately characterize the transport of VA-SNEDDS on the Caco-2 cell monolayer, we employed a multiscale imaging strategy. In this study, the lipophilic fluorescent dyes DiO and DiI were employed as drug surrogates. After incubating DiO/DiI-VA-SNEDDS on the apical side of the Caco-2 cell monolayers for 4 h, fluorescence was detected in both the apical and basolateral HBSS solutions. Co-localization of red (DiI) and green (DiO) signals was observed in the apical compartment following the administration of dual-labeled nanoemulsions, confirming successful encapsulation of both dyes within the same nanocarriers. Notably, fluorescence co-localization was also detected in the basolateral HBSS solutions, indicating that the association between the co-loaded dyes was maintained during transport, thereby demonstrating the preservation of the delivery system's functional integrity (Figure 4B). Owing to the diffraction-limited resolution (around 200 nm) of confocal laser scanning microscopy (CLSM), the observed fluorescent puncta (around 2 µm) represent diffraction-limited spots formed by the superposition of signals from multiple adjacent nanoparticles (Betzig et al. 2006; Schermelleh et al. 2010), reflecting the potential local enrichment or dynamic aggregation state of nanoparticles in biological fluids (Mahmoudi et al. 2011).

To directly evaluate nanostructural integrity at the single-particle level, we performed TEM analysis. The samples were diluted with deionized water to disperse reversible loose aggregates. Furthermore, TEM analysis revealed that the MOR-VA-SNEDDS formed well-defined (approximately 20 nm), uniformly distributed spherical droplets on the apical side of the Caco-2 cell monolayer within HBSS solution. After 4 h of co-incubation with Caco-2 cells, similar spherical droplets were observed on the basolateral side, although some nanoemulsions exhibited a slight increase in size (approximately 50 nm), further supporting the structural stability of VA-SNEDDS during gastrointestinal transit (Figure 4C). This direct morphological evidence demonstrates that VA-SNEDDS traverses the intestinal epithelial barrier while maintaining an intact nanostructure and undergoes a specific size transformation, suggesting an interaction with endogenous biomolecules such as the apolipoprotein ApoB-48.

Based on existing knowledge regarding the intestinal transport of nanoparticles (Wang and Luo 2019), it is postulated that endocytic vesicles containing MOR-VA-SNEDDS fuse with apical early endosomes (EE), after which specific nanoparticles are exocytosed *via* alternative transcytotic pathways at the basolateral side. Additionally, there was the lower level of VA-SNEDDS observed on the basolateral side compared to the apical side, which may be attributed to the following pathways: (1) some VA-SNEDDS may be recycled back to the apical membrane from early endosomes (Sahay et al. 2010); or (2) a portion may be trafficked to late endosomes (LE) and ultimately to lysosomes, where degradation by hydrolytic enzymes occurs (Guo et al. 2013). The ‘easy entry, difficult exit’ phenomenon is a typical characteristic of nanoparticles crossing intestinal epithelial cells (Ejazi et al. 2023; Wang and Luo 2019). Nevertheless, our results demonstrated that the VA-SNEDDS could mimic CMs by maintaining structural integrity during translocation across the gastrointestinal barrier.

#### 3.3.3. The nano-biological self-assembly process of VA-SNEDDDS in Caco-2 cells

Apolipoprotein B-48 (ApoB-48), a key protein in CMs assembly, remains attached to the CMs remnants following hydrolysis by systemic lipases (Masuda et al. 2025). Therefore, ApoB-48 is considered an ideal marker for studying the *in vivo* metabolism of CMs. The expression of the ApoB-48 protein on the surface of MOR-VA-SNEDDS after exocytosis was detected by the WB analysis. The results showed a clear ApoB-48 signal in the eluate on the basolateral side, with grayscale values confirming the expression of the target protein (Figure 4D and E). Combined with those of microscopic imaging, these findings further confirmed that MOR-VA-SNEDDS interacted with ApoB-48 in the endoplasmic reticulum (ER) at the nanobio level and participated in CMs assembly during ER/Golgi (GC) trafficking. Thus, the large-sized particles observed under the microscope may represent novel nanoemulsions formed after CMs assembly.

#### 3.3.4. Effect of MOR-VA-SNEDDS on the barrier function of Caco-2 cells

To investigate whether the formulation affected the integrity of the cellular barrier, we performed a sodium fluorescein permeability assay on Caco-2 monolayers after the transport experiment. As shown in Table 2, compared to the HBSS control group, the Papp of sodium fluorescein in the MOR-VA-SNEDDS-treated group showed no statistically significant increase at 0.5, 1, and 2 h (*p* > 0.05). After 2 h of treatment, the Papp value in the MOR-VA-SNEDDS group was (6.43 ± 0.30) × 10^−7^ cm/s, which was well below the specified threshold of 1 × 10^−6^ cm/s. These results demonstrated that the Caco-2 monolayer model maintained a tight and intact cellular monolayer after the experiment, with permeability meeting the required standards.

**Table 2.** Apparent permeability coefficient (Papp, × 10^−7^ cm/s) of sodium fluorescein across Caco-2 monolayers (*n* = 3).

Sample	0.5 h	1 h	2 h
Control (HBSS)	3.71 ± 0.17	4.34 ± 0.18	5.61 ± 0.31
MOR-VA-SNEDDS	3.79 ± 0.20	4.78 ± 0.27	6.43 ± 0.30

#### 3.3.5. Plasma localization of VA-SNEDDS

The aforementioned experiments confirmed that nanoemulsions can be excreted intact by intestinal epithelial cells. To further verify their structural integrity after penetrating the gastrointestinal barrier and entering the circulation, we conducted *in vivo* experiments to observe the distribution of DiO/DiI-loaded nanoemulsions in rat blood. Following administration of the mixture of DiO-VA-SNEDDS and DiI-VA-SNEDDS, the red and green fluorescence signals were distinctly separated (Figure 4F), indicating an absence of co-localization between DiO and DiI in the circulation. In contrast, DiO/DiI-VA-SNEDDS exhibited overlapping red and green fluorescence signals in the blood, confirming the effective co-encapsulation of both dyes without leakage. These findings further confirmed that VA-SNEDDS could achieve efficient intracellular transport and subsequent extracellular release without compromising nanoparticle integrity, which is essential for both intestinal barrier penetration and systemic targeted delivery.

### 3.4. *In vivo* hepatic targeting and cellular localization of VA-SNEDDS

**Figure 5.**
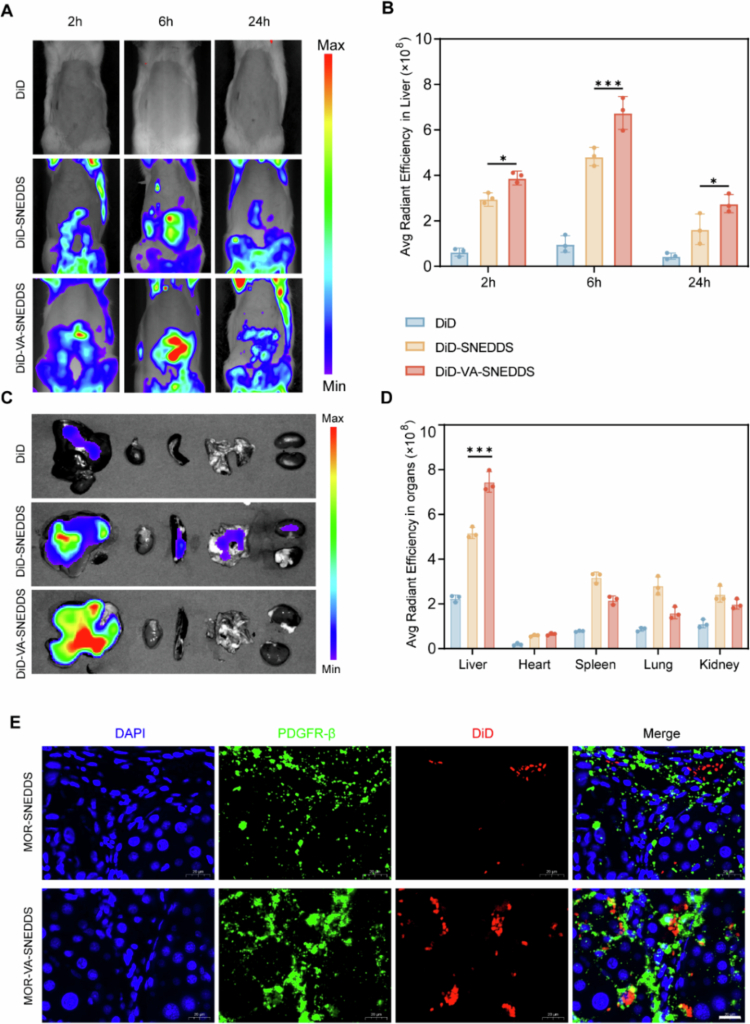
VA-SNEDDS selectively accumulates in the liver and hepatic stellate cells. (a) Representative images of near-infrared fluorescence detection in liver fibrosis model rats at various time points. (b) Statistical analysis of fluorescence intensity in the livers of liver fibrosis model rats (*n* = 3). (c) Representative images of near-infrared fluorescence detection in major organs. (d) Statistical analysis of fluorescence intensity in major organs (*n* = 3). (e) The distribution of different DiD-labeled formulations in HSCs within fibrotic livers. HSCs were determined by immunofluorescence analysis of PDGFR-β (green fluorescence). Scale: 20 μm. The values represent mean ± SD; *p < 0.05, ***p < 0.001.

To further substantiate the enhanced hepatic targeting capability of VA-SNEDDS *in vivo*, we investigated the biodistribution of DiD-labeled VA-SNEDDS and monitored the temporal fluorescence signal in rats through real-time imaging. Within the first two hours following administration, the fluorescence signal in fibrotic rats gavaged with DiD-SNEDDS or DiD-VA-SNEDDS was predominantly localized to the abdominal region. The VA group subsequently exhibited a progressive intensification of fluorescence within the hepatic region, reaching its peak at six hours post-administration (Figure 5A). Semi-quantitative analysis revealed that the mean radiant efficiency of DiD-SNEDDS group in the liver was 4.9-fold greater than that observed for free DiD, while the VA group exhibited an impressive 6.9-fold increase (Figure 5B). At 24 h post-administration, *ex vivo* organ imaging and corresponding semi-quantitative data demonstrated that the inclusion of VA significantly enhanced the accumulation of nanoemulsions in hepatic tissue while concomitantly reducing their distribution to other organs (Figure 5C and D).

To verify the specific uptake of VA-SNEDDS by aHSCs in liver fibrosis, tissue immunofluorescence staining was performed for colocalization analysis. The results showed that the DiD signals (red) in the VA-SNEDDS overlapped with those in the activated HSC receptor PDGFR-β (green), indicating that VA-SNEDDS were located mainly within aHSCs (Figure 5E). In fibrotic liver tissue, the overlapping area between PDGFR-β-positive aHSCs and DiD-VA-SNEDDS was larger than that of DiD-SNEDDS, suggesting that the VA modification could remarkably enhance the live targeting ability of SNEDDS to aHSCs. Although lipid-associated nanoparticles are capable of passively delivering drug components to the liver because of their lipophilicity and easy recognition by macrophages (Kulkarni et al. 2019). In fibrotic livers, aHSCs upregulate the RBP receptor (Luo et al. 2023), which is beneficial for improving the aHSCs-targeting efficiency of VA-SNEDDS through the VA and RBP receptor-mediated binding. The results of this study confirmed the selective accumulation of VA-SNEDDS in aHSCs within fibrotic livers, demonstrating its potential as a liver-targeted therapeutic strategy.

### 3.5. Pharmacokinetic behavior of MOR-VA-SNEDDS

**Figure 6.**
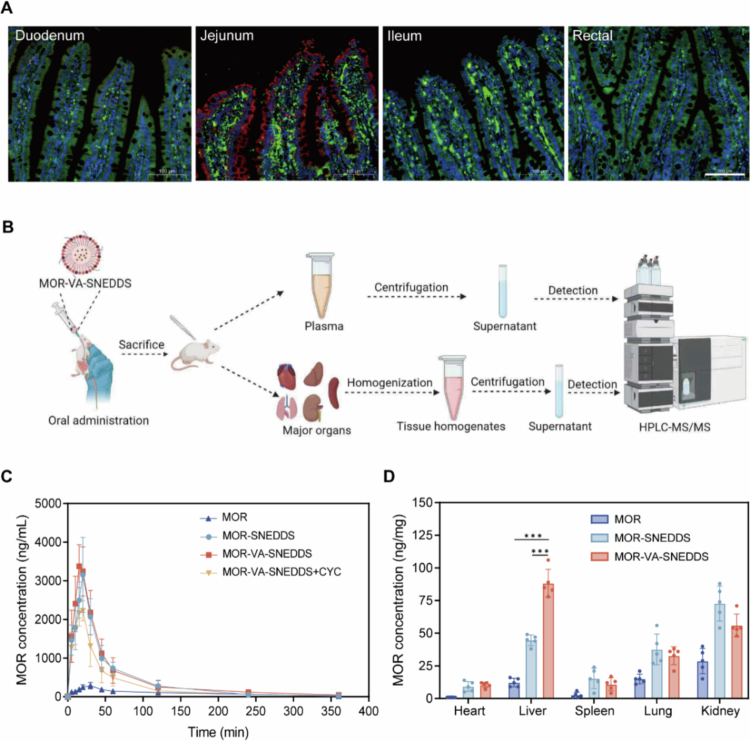
Pharmacokinetics and biodistribution of MOR-VA-SNEDDS. (a) The absorption of VA-SNEDDS in different intestinal segments following oral administration of DiD-VA-SNEDDS in rats. Green fluorescence represents F-actin, and red fluorescence represents DiD. Scale: 100 μm. (b) Schematic diagram of the pharmacokinetic study in rats after oral administration. (Created with BioRender.com). (c) Average blood drug concentration‒time profiles for rats orally administered suspensions of MOR, MOR-SNEDDS, MOR-VA-SNEDDS, and MOR-VA-SNEDDS + cyclohexylamine (*n* = 5). (d) Drug content in major organs of rats orally administered suspensions of MOR, MOR-SNEDDS, and MOR-VA-SNEDDS 30 min post-administration (*n* = 5). The values represent mean ± SD; ***p < 0.001.

In order to investigate the absorption site of VA-SNEDDS in the intestinal tract, DiD-VA-SNEDDS was gavaged into rats, and the intestine was collected and observed by a fluorescence microscope. As shown in Figure 6A, the red fluorescence observed in the jejunum was much higher than that in the duodenum, ileum and rectal and substantial red fluorescence had been transported across the intestinal epithelium, which inferred that VA-SNEDDS was mainly absorbed from the jejunum. Subsequently, HPLC-MS/MS was employed to quantify drug concentrations in plasma after oral administration of MOR-VA-SNEDDS in rats (Figure 6B). Based on the mean plasma concentration‒time curve, the plasma concentrations of MOR in the MOR-VA-SNEDDS group at all time points in rats were significantly higher than those of the MOR group. The pharmacokinetic parameters (Table 3) showed that the plasma concentration of MOR for the MOR-VA-SNEDDS group reached its peak at 19.17 ± 2.04 min (C_max_ = 3279.59 ± 852.72 μg/L), which was 11.30 times higher than that of the MOR suspension group (C_max_ = 289.80 ± 81.67 μg/L). The MOR-VA-SNEDDS group achieved its peak plasma concentration at 17.50 ± 2.74 min (C_max_ = 3723.01 ± 302.18 μg/L), which was 12.80 times higher than that of the MOR suspension group. Furthermore, the area under the plasma concentration‒time curve (AUC_0-t_) of the MOR-VA-SNEDDS group was 171.00 ± 34.48 mg/L*min, which was significantly higher than that of the MOR group (33.80 ± 6.21 mg/L*min) (*p* < 0.001) (Figure 6C). Interestingly, the pharmacokinetic parameters did not differ significantly between the MOR-SNEDDS and MOR-VA-SNEDDS groups, revealing that the incorporation of VA had no effect on the oral absorption of the SNEDDS. These results illustrated that VA-SNEDDS could markedly improve the oral bioavailability of MOR, which may be attributed to the ability of nanoemulsions to readily diffuse through the intestinal mucus layer and enter the brush border membrane of enterocytes. Additionally, VA-SNEDDS is mainly absorbed at the jejunum (Figure 6A), where exhibit the high expression of P-glycoprotein (P-gp), a drug efflux pump, (Englund et al. 2006), and MOR is the substrate of P-gp (Teng et al. 2021). Therefore, Cremophor RH 40 with in VA-SNEDDS, a P-gp inhibitor, suppresses the MOR efflux by P-gp, consequently enhancing MOR absorption in the intestinal tract. In order to verify the lymphatic transport of MOR-VA-SNEDDS, CYC, a protein synthesis inhibitor, was adopted to block lymphatic transport because it can effectively suppress the secretion of CMs in intestinal cells without affecting other active or passive absorption pathways (Ryšánek et al. 2021). As shown in Figure 6C, with the co-administration of CYC, the C_max_ of MOR-VA-SNEDDS decreased by 34.84%, and the AUC_0-t_ decreased by 35.67%. This result inferred that lymphatic transport played a vital role in the oral absorption of MOR-VA-SNEDDS.

**Table 3.** Pharmacokinetic parameters of MOR after oral administration of MOR suspension, MOR-SNEDDS, MOR-VA-SNEDDS, and MOR-VA-SNEDDS + CYC in rats (*n* = 5).

Parameter	MOR suspension	MOR-SNEDDS	MOR-VA-SNEDDS	MOR-VA-SNEDDS + CYC
AUC_(0__-t)_ (mg/L*min)	33.80 ± 6.21	153.33 ± 19.96	171.00 ± 34.48***	110.25 ± 9.54^#^
AUC_(0-∞)_ (mg/L*min)	34.70 ± 5.85	155.17 ± 20.23	177.67 ± 33.10***	112.75 ± 8.97^##^
*t*_max_ (min)	31.00 ± 8.94	19.17 ± 2.04	17.50 ± 2.74	15.00 ± 4.08
*C*_max_ (μg/L)	289.80 ± 81.67	3279.59 ± 852.72	3723.01 ± 302.18***	2426.25 ± 142.43^###^

Notes: Data are presented as mean ± SD. ***p < 0.01, compared with the MOR suspension group. ^#^ *p *< 0.05, ^##^ *p *< 0.01, ^###^ *p *< 0.001, compared with the MOR-VA-SNEDDS group.

### 3.6. *In vivo* biodistribution of MOR-VA-SNEDDS

In order to quantitatively assess the liver-targeting ability of MOR-VA-SNEDDS, the MOR concentration in major organs was determined by LC-MS/MS. As shown in Figure 6D, in the MOR group, the MOR concentration in the kidney (28.36 ± 8.69 ng/mg) was higher than that in the liver (12.12 ± 2.93 ng/mg), indicating that the kidney is the primary distribution organ of MOR. In the MOR-SNEDDS and MOR-VA-SNEDDS groups, the MOR concentration in all the organs significantly increased compared to the MOR group (*p* < 0.001). Interestingly, there was the highest accumulation in the liver for MOR-VA-SNEDDS group. The MOR concentration in the liver for MOR-VA-SNEDDS reached 88.29 ± 9.58 ng/mg, which was approximately 7.28 and 2.00 times that of the MOR and MOR-SNEDDS, respectively. These results further demonstrated that MOR-VA-SNEDDS possessed a good liver-targeting ability.

### 3.7. Antifibrotic activity of MOR-VA-SNEDDS

**Figure 7.**
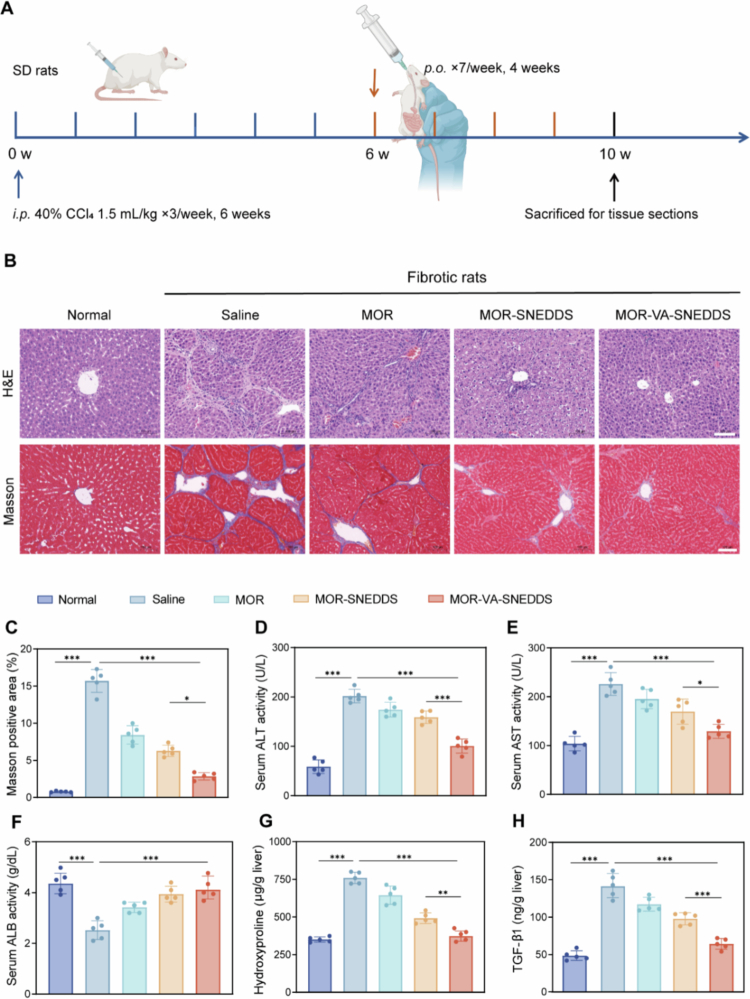
Antifibrotic activity of MOR-VA-SNEDDS in liver fibrosis model rats. (a) Schematic diagram showing the establishment of animal models and drug treatment studies. (b) Representative histological sections of liver tissue stained with H&E (200×) and Masson (100×). (c) Semi-quantitative analysis of Masson-positive area in the tissue slices (*n* = 5). (d–f) Serum levels of ALT (d), AST (e), and ALB (f) in rats with hepatic fibrosis following treatment with MOR-VA-SNEDDS (*n* = 5). (g, h) Hepatic concentration of hydroxyproline (g) and TGF-β1 (h) in rats with liver fibrosis after treatment with MOR-VA-SNEDDS (*n* = 5). The values represent mean ± SD; *p < 0.05, **p < 0.01, ***p < 0.001.

To evaluate the therapeutic effect of MOR-VA-SNEDDS on hepatic fibrosis in this study, we used a rat model in which fibrosis was induced by continuous intraperitoneal injection of CCl_4_, and the experimental procedure is shown in Figure 7A. The therapeutic effect of MOR-VA-SNEDDS was evaluated by immunofluorescence staining and biochemical indicators. After H&E and Masson staining, the livers of normal rats showed intact hepatic lobular structure without hepatocyte inflammation or edema (Ishak-score 0). In contrast, liver tissue sections from the rats with liver fibrosis showed severe centrilobular necrosis, collagen fiber deposition, an enlarged portal area, and fibrous bridging formation (Ishak-score 4). Compared with the saline group, the MOR, MOR-SNEDDS, and MOR-VA-SNEDDS treatments all showed different degrees of inhibition against liver fibrosis (Figure 7B). Among them, liver sections of rats in the MOR-VA-SNEDDS group showed a significant reduction in fibroplasia, with negligible fibrous deposition in the portal ductal area (Ishak-score 1). The results of the semiquantitative analysis of Masson staining further verified this trend (Figure 7C), suggesting that MOR-VA-SNEDDS has an obvious advantage in alleviating hepatic fibrosis.

To quantitative evaluate liver function, the serum levels of biomarkers, including ALT, AST and ALB, were determined (Kwo et al. 2017). As shown in Figure 7D‒F, the serum levels of ALT and AST were significantly higher in fibrotic rats than in normal rats, while the serum level of ALB was significantly lower. After treatment with MOR suspension or MOR-SNEDDS, all of the above abnormal indexes were reversed to a certain degree extent. Excitingly, the serum levels of liver function biomarkers in the MOR-VA-SNEDDS group were close to those in the normal group. Additionally, the average concentrations of HYP and TGF-β1, indicators of liver fibrosis (Gressner et al. 2007), in the liver within the saline-treated rats with liver fibrosis were significantly higher than that of normal rats. Treatment with MOR suspension or MOR-SNEDDS reduced the liver expression of HYP and TGF-β1i to some degrees. Moreover, treatment with MOR-VA-SNEDDS was found to restore the levels of these liver fibrosis mediators to near-normal values (Figure 7G and H). In summary, treatment of MOR-VA-SNEDDS remarkably alleviated the hepatic fibrosis process in rats, validating its potential in hepatic fibrosis-targeting therapy.

## 4. Conclusion

In this study, we successfully developed an orally administrable vitamin A-functionalized self-assembly nanoemulsion for the targeted delivery of morin to aHSCs in liver fibrosis. The MOR-VA-SNEDDS effectively overcame the challenge of intestinal degradation, displayed efficient translocation across the intestinal epithelium and subsequently entered into systemic circulation *via* the lymphatic pathway. This delivery system achieved precise accumulation in aHSCs within fibrotic livers *via* both the retinol-binding protein receptor-mediated pathway and the direct interaction of VA with other VA-binding proteins on the cell membrane.

In a CCl_4_-induced rat model of hepatic fibrosis, MOR-VA-SNEDDS exhibited potent anti-fibrotic efficacy, significantly attenuated ECM deposition, reduced hydroxyproline and TGF-β1 levels, and restoring liver function. These findings highlight the high translocation efficiency and therapeutic potential of this orally delivered, aHSC-targeted platform as a promising strategy for the long-term treatment of liver fibrosis.

## Supplementary Material

Supplementary Material ARRIVE_Guidelines_Checklist.pdf

## Data Availability

The data presented in this study are available from the corresponding author, Q.Z., upon reasonable request.
